# Symmetry breaking of highly symmetrical nanoclusters for triggering highly optical activity

**DOI:** 10.1016/j.fmre.2022.03.007

**Published:** 2022-03-29

**Authors:** Xiao Wei, Hao Li, Honglei Shen, Chuanjun Zhou, Shuxin Wang, Xi Kang, Manzhou Zhu

**Affiliations:** Department of Chemistry and Centre for Atomic Engineering of Advanced Materials, Key Laboratory of Structure and Functional Regulation of Hybrid Materials of Ministry of Education, Institutes of Physical Science and Information Technology and Anhui Province Key Laboratory of Chemistry for Inorganic/Organic Hybrid Functionalized Materials. Anhui University, Hefei 230601, China

**Keywords:** Atomically precise nanocluster, Symmetry breaking, Enantioseparation, Circularly polarized luminescent, Photoluminescence

## Abstract

Developing new approaches to fulfill the enantioseparation of nanocluster racemates and construct cluster-based nanomaterials with optical activity remains highly desired in cluster science, because it is an essential prerequisite for fundamental research and extensive applications of these nanomaterials. We herein propose a strategy termed “active-site exposing and partly re-protecting” to trigger the symmetry breaking of highly symmetrical nanoclusters and to render cluster crystals optically active. The vertex PPh_3_ of the symmetrical Ag_29_(SSR)_12_(PPh_3_)_4_ (SSR = 1, 3-benzenedithiol) nanocluster was firstly dissociated in the presence of counterions with large steric hindrance, and then the exposed Ag active sites of the obtained Ag_29_(SSR)_12_ nanocluster were partly re-protected by Ag^+^, yielding an Ag_29_(SSR)_12_-Ag_2_ nanocluster with a symmetry-breaking construction. Ag_29_(SSR)_12_-Ag_2_ followed a chiral crystallization mode, and its crystal displayed strong optical activity, derived from CD and CPL characterizations. Overall, this work presents a new approach (i.e., active-site exposing and partly re-protecting) for the symmetry breaking of highly symmetrical nanoclusters, the enantioseparation of nanocluster racemates, and the achievement of highly optical activity.

## Graphical Abstract

**Figure:**
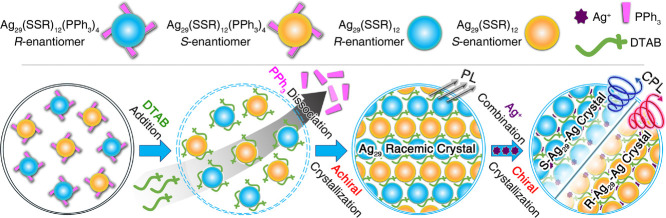

## 1 Introduction

Chirality has long been one of the most important research topics since it is an amazing phenomenon ubiquitous in life, nature, and the universe ranging from nanoscale molecules (e.g., proteins, sugars, and DNA) to mater-scale substances (e.g., eyes, shells, and flowers), and to the vast galaxy [1], [2], [3]. As one of the most appealing characteristics of nanostructures, optical activity, including circular dichroism (CD), circular polarized luminescence (CPL), and vibrational circular dichroism (VCD), has attracted much attention and been considered as the prerequisite to exploit chirality-related applications [4,5]. The study of chirality dated back to 1811 when the optical activity was observed in asymmetric quartz crystal by François Arago, after which tremendously experimental and theoretical efforts were made [6], [7], [8]. In nanoscience, the optical activity is generally achieved either by constructing metal nanoparticle-based assemblies in chiral arrangements or by the conjugation of metal nanoparticles with peripheral chiral molecules [9], [10], [11]. Indeed, the large-sized nanoparticle itself without chiral stabilizers is almost optically inactive in light of its homogeneous and symmetrical packing in the metallic kernel. In vivid contrast, metal nanoclusters, routinely described as ultrasmall metal nanoparticles [12], [13], [14], [15], [16], [17], [18], [19], [20], [21], [22], [23], have been exploited as ideal platforms to investigate the intrinsic chirality of metallic kernels, owing to their plentiful kernel constructions and kernel-surface bonding environments [24], [25], [26].

The chirality of nanoclusters mainly originates from three aspects: (i) chirality in the metallic kernel, (ii) chirality in the metal-ligand interface, and (iii) chirality in the peripheral carbon group [27], [28], [29], [30], [31], [32], [33], [34], [35], [36], [37], [38]. Among these aspects, the first aspect (i.e., chiral metallic kernel) has attracted much scientific interest since it not only represents the most intrinsic character in analyzing the origin of chirality in nanomaterials, but also exists in the other two aspects due to the transmission effect via intracluster interactions [39]. Besides, aside from nanoclusters with chiral peripheral ligands, most structurally asymmetrical nanoclusters with achiral ligands are racemic in their solutions and crystals [40,41]. In this context, the chiral separation of these racemic clusters is an essential prerequisite for their fundamental research and extensive applications. Although several approaches (e.g., high-performance liquid chromatography separation and chiral self-assembly) have been exploited to separate cluster enantiomers from their racemates [42], [43], [44], [45], [46], [47], the enantioseparation has only been accomplished in limited examples. New approaches to fulfill the chiral resolution of nanocluster racemates and construct cluster-based nanomaterials with optical activity remain highly desired in cluster science.

**Scheme 1:**
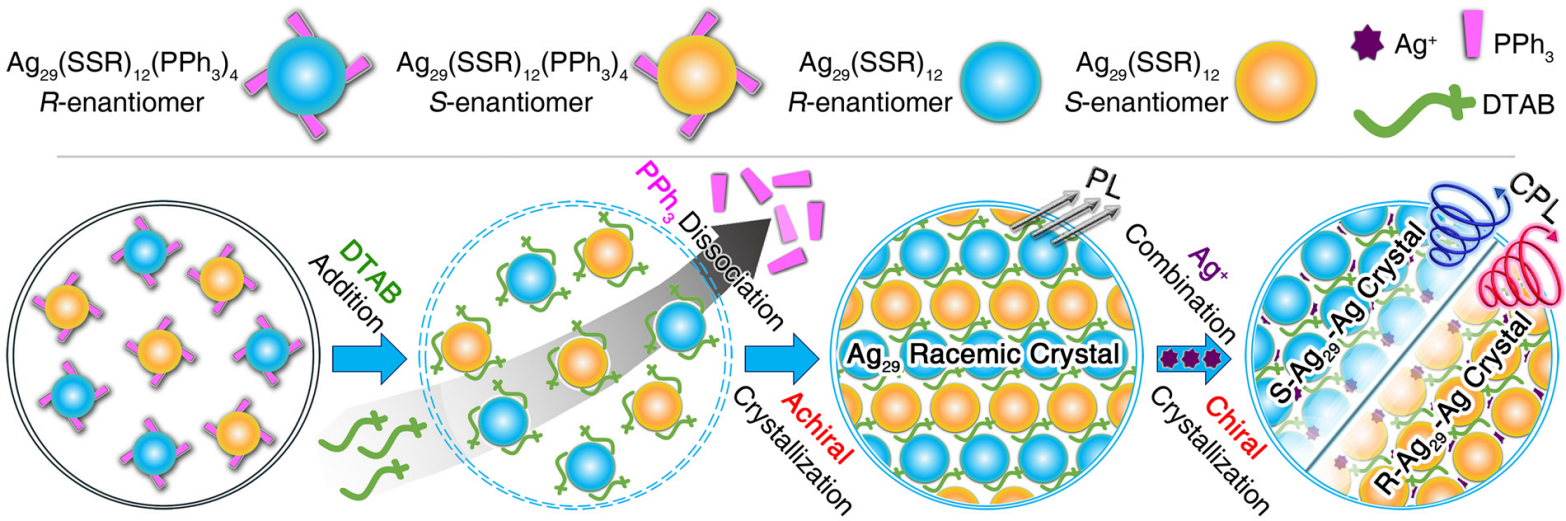
**Illustration of the “active-site exposing and partly re-protecting” strategy for triggering the symmetry breaking of highly symmetrical nanoclusters and rendering cluster crystals optically active**.

Herein, a strategy termed “active-site exposing and partly re-protecting” was proposed to trigger the symmetry breaking of highly symmetrical nanoclusters and to render cluster crystals optically active (Scheme 1). The Ag_29_(SSR)_12_(PPh_3_)_4_ (Ag_29_-PPh_3_ for short; SSR = 1,3-benzenedithiol) nanocluster was highly symmetrical with four Ag-PPh_3_ vertex units. The introduction of counterions with large steric hindrance to the nanocluster system induced the dissociation of vertex PPh_3_ and the generation of Ag_29_(SSR)_12_ (Ag_29_ for short) with exposed surface Ag active sites. Furthermore, the Ag^+^ addition triggered the re-protection of partly exposed Ag sites on the Ag_29_ nanocluster surface, yielding the Ag_29_(SSR)_12_-Ag_2_ (Ag_29_-Ag for short) nanocluster with a symmetry-breaking construction. The cluster crystals of both Ag_29_-PPh_3_ and Ag_29_ clusters were racemic; by comparison, the crystallization of Ag_29_-Ag followed a chiral mode, accomplishing the enantioseparation of nanocluster racemates. Accordingly, the crystal of the symmetry-breaking Ag_29_-Ag cluster displayed strong optical activity, derived from CD and CPL characterizations.

The DTAB addition-induced PPh_3_ dissociation rendered vertex active Ag of the Ag_29_-PPh_3_ nanocluster exploring, while the obtained Ag_29_ cluster still adhered to the racemic crystallization. Besides, the Ag^+^ addition triggered the re-protection of partly exposed Ag active sites and the symmetry breaking of the nanocluster, and the crystallization of Ag_29_-Ag cluster entities follows a chiral mode.

## 2 Experimental methods

### 2.1 Materials

All following reagents were purchased from Sigma-Aldrich and used without further purification, including silver nitrate (AgNO_3_, 99%, metal basis), triphenylphosphine (PPh_3_, 99%), 1,3-benzene dithiol (SSR, 99%), sodium borohydride (NaBH_4_, 99%), dodecyltrimethylammonium bromide (CH_3_(CH_2_)_10_CH_2_-N-(CH_3_)_3_Br, DTAB, 98%), tetramethylammonium bromide ((CH_3_)_4_NBr, TMAB, 98%), tetramethylammonium bromide ((C_4_H_9_)_4_NBr, TBAB, 98%), silver acetate (CH_3_COOAg, 99%), methylene chloride (CH_2_Cl_2_, HPLC, Sigma-Aldrich), methanol (CH_3_OH, HPLC grade), N,N-dimethylformamide (DMF, HPLC grade), and ethyl ether ((CH_3_CH_2_)_2_O, HPLC grade).

### 2.2 Synthesis of Ag_29_(SSR)_12_(PPh_3_)_4_ (Ag_29_-PPh_3_)

The preparation of Ag_29_-PPh_3_ was based on the reported method of the Bakr group [48]. The counterion of Ag_29_-PPh_3_ was Na^+^.

### 2.3 Nanocluster transformation from Ag_29_-PPh_3_ to Ag_29_(SSR)_12_ (Ag_29_)

30 mg of the Ag_29_-PPh_3_ crystal was dissolved in 10 mL of DMF, and 50 mg of DTAB was added under vigorous stirring. After 30 min, the organic layer was separated to produce the Ag_29_ nanocluster, which was used for crystallization directly. The yield was 95% based on the Ag element (calculated from the Ag_29_-PPh_3_).

### 2.4 Nanocluster transformation from Ag_29_ to Ag_29_(SSR)_12_-Ag_2_ (Ag_29_-Ag)

30 mg of the Ag_29_ crystal was dissolved in 10 mL of DMF, and 3 mg of CH_3_COOAg was added under vigorous stirring. After 30 min, the organic layer was separated and poured into 200 mL of CH_2_Cl_2_. The precipitate was collected to produce the Ag_29_-Ag nanocluster. The yield was 90% based on the Ag element (calculated from the Ag_29_). Of note, the crystal analysis demonstrated that the Ag_29_-Ag crystal contained both Ag_29_-Ag and Ag_29_ nanoclusters. The counterions of both Ag_29_ and Ag_29_-Ag were DTAB.

### 2.5 Crystallization of the Ag_29_ nanocluster series

Single crystals of these Ag_29_ nanoclusters were cultivated at 15 °C by vapor diffusing the ethyl ether into a DMF solution of the cluster. After 2 weeks, red crystals were collected, and the structures of these Ag_29_ nanoclusters were determined.

### 2.6 Preparation of nanocluster crystalline films

The concentration of the DMF/CH_2_Cl_2_ (1:9 of the volume ratio) solution of these Ag_29_ nanoclusters was set as 30 mg/mL and then the solution was filtered with a 0.2 µm syringe filter. The solutions were stored for 12 h before use. 50 µL of the solutions were dropped onto a quartz substrate, and spin-coated (using LAURELL WS-650MZ-23NPPB) at 1000 rpm for 60 s. The cluster-impregnated quartz substrate was dried in the air for 12 h before the optical property characterization.

### 2.7 Characterizations

The optical absorption spectra of nanoclusters were recorded using an Agilent 8453 diode array spectrometer.

The photoluminescence (PL) spectra were measured on a FL-4500 spectrofluorometer with the same optical density.

Electrospray ionization mass spectrometry (ESI-MS) measurements were performed by a Waters XEVO G2-XS QTof mass spectrometer. The sample was directly infused into the chamber at 5 μL/min. For preparing the ESI samples, nanoclusters were dissolved in DMF (1 mg/mL) and diluted (*v*/*v* = 1:1) by CH_3_OH.

^31^P nuclear magnetic resonance (NMR) spectra were acquired using a Bruker 600 Avance III spectrometer equipped with a Bruker BBO multinuclear probe (BrukerBioSpin, Rheinstetten, Germany).

The circularly polarized luminescence (CPL) spectra of nanoclusters were recorded using a JASCO CPL-300 instrument. For CPL measurements, the parameters were set as follows: scanning speed of 200 nm/min, response time of 2 s, band width of 10 nm, accumulations of 6.

The circular dichroism (CD) spectra were measured on a JASCO J-1500 circular dichroism spectrophotometer. The solid samples were measured with a DRCD-574 solid samples accessories, using an integrating sphere to detect the diffuse reflectance of samples.

### 2.8 X-Ray crystallography

The data collection for single-crystal X-ray diffraction (SC-XRD) of all nanocluster crystal samples was carried out on Stoe Stadivari diffractometer under nitrogen flow, using graphite-monochromatized Cu Kα radiation (λ = 1.54186 Å). Data reductions and absorption corrections were performed using the SAINT and SADABS programs, respectively. The structure was solved by direct methods and refined with full-matrix least squares on F^2^ using the SHELXTL software package. All non-hydrogen atoms were refined anisotropically, and all the hydrogen atoms were set in geometrically calculated positions and refined isotropically using a riding model. All crystal structures were treated with PLATON SQUEEZE. The diffuse electron densities from these residual solvent molecules were removed. The CCDC number of the racemic Ag_29_(SSR)_12_ nanocluster is 2071574. The CCDC number of the chiral Ag_29_(SSR)_12_-Ag_2_ (*S* enantiomer) nanocluster is 2071575. The CCDC number of the chiral Ag_29_(SSR)_12_-Ag_2_ (*R* enantiomer) nanocluster is 2071643. The CCDC number of Ag_29_(SSR)_12_(PPh_3_)_4_ in the presence of TMAB is 2150072. The CCDC number of Ag_29_(SSR)_12_(PPh_3_)_4_ in the presence of TBAB is 2150121.

## 3 Results and discussion

The Ag_29_-PPh_3_ nanocluster was prepared using the previously reported procedure [48]. The introduction of DTAB (N,N,N-trimethyl-1-dodecanaminium bromide) induced the transformation from Ag_29_-PPh_3_ to Ag_29_, and the Ag_29_-Ag nanocluster was obtained via anchoring Ag^+^ onto the Ag_29_ nanocluster surface (see Experimental Method for more details). Only two Ag^+^ ions could be introduced onto the nanocluster surface, while the Ag_29_(SSR)_12_-Ag*_x_* nanoclusters with *x* = 1, 3, 4 were absent. Such a tendency might result from the tunable chemical reactivity of the Ag_29_(SSR)_12_ framework in reacting with the introduced Ag^+^ ions. The bare Ag_29_(SSR)_12_ could react with Ag^+^ with a high degree of activity, while the Ag_29_-Ag showed no activity to further react with Ag^+^. That is, the addition of Ag^+^ onto the Ag_29_(SSR)_12_ framework might passivate the corresponding nanocluster. ESI-MS measurement was performed to determine the molecular formula of these Ag_29_ nanoclusters (Fig. S1). The ESI-MS spectrum of Ag_29_-PPh_3_ exhibited five separated signals, corresponding to the [Ag_29_(SSR)_12_(PPh_3_)*_n_*]^3−^ where *n* ranged from 0 to 4 (Fig. S1a,b). The existence of these five peaks was in agreement with the previously reported “dissociation-aggregation pattern” of the PPh_3_ ligands in the nanocluster [49]. The mass spectrum of Ag_29_ showed an intense mass peak that matched the [Ag_29_(SSR)_12_]^3−^. All PPh_3_ ligands were dissociated from the Ag_29_ surface after the DTAB addition since no peak of [Ag_29_(SSR)_12_(PPh_3_)*_n_*]^3−^ (*n* = 1-4) was observed (Fig. S1c). The Ag_29_-Ag displayed two mass peaks, as shown in Fig. S1d, and the excellent match of the experimental and simulated isotope patterns demonstrated that these two peaks matched the [Ag_29_(SSR)_12_]^3−^ and [Ag_29_(SSR)_12_-Ag]^2−^, respectively. The mass signal of [Ag_29_(SSR)_12_-Ag]^2−^ verified the capture of Ag^+^ onto the Ag_29_(SSR)_12_ surface. However, the mass peak of [Ag_29_(SSR)_12_-Ag_2_]^1−^ was absent, resulting from the weak interactions between the Ag_29_(SSR)_12_ framework and Ag^+^ ions. Besides, the PPh_3_ dissociation among the conversion from Ag_29_-PPh_3_ to Ag_29_ and Ag_29_-Ag was further verified by the ^31^P NMR measurement, where the 26.20 ppm signal of Ag_29_-PPh_3_ disappeared in the latter two nanoclusters (Fig. S2).

**Fig. 1:**
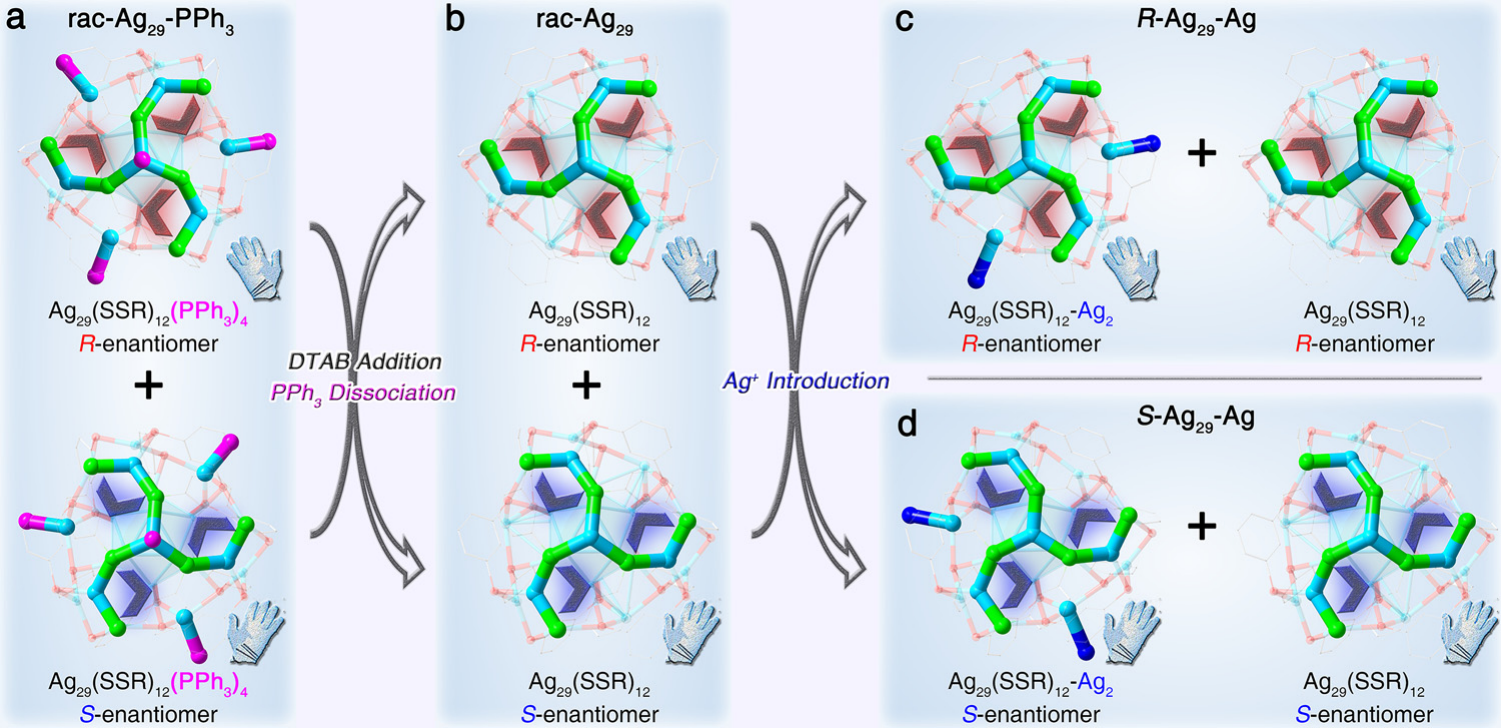
**Atomically precise structures of the Ag_29_ nanocluster series**. (a) The racemic Ag_29_ nanocluster enantiomers. (b) The racemic Ag_29_ nanocluster enantiomers. (c) The chiral Ag_29_-Ag nanoclusters (*R* enantiomer). (d) The chiral Ag_29_-Ag nanoclusters (S enantiomer). Color legends: blue/light blue sphere, Ag; red spher e, S; magenta sphere, P; grey sphere, C. For clarity, all H atoms are omitted.

The structural comparisons of these Ag_29_ nanoclusters are shown in Fig. 1 and S2–S4. The Ag_29_-PPh_3_ contained an icosahedral Ag_13_ kernel that was stabilized by four Ag_3_(SR*)_6_, where two SR* make up a SSR, to generate an Ag_25_(SSR)_12_ framework. The four terminals of Ag_25_(SSR)_12_ were further capped by Ag-PPh_3_ units, giving rise to the overall structure of the highly symmetrical Ag_29_-PPh_3_ (Fig. 1a and S3). Upon the DTAB-addition induced conversion from Ag_29_-PPh_3_ to Ag_29_, the PPh_3_ ligands on the Ag_29_-PPh_3_ surface were dissociated while the overall configuration of nanocluster remained highly symmetrical (Fig. 1b and S4). Such a PPh_3_ dissociation was proposed to result from the competition effect between PPh_3_ and DTAB ― the PPh_3_ ligands followed a “dissociation-aggregation pattern” on the Ag_29_ nanocluster surface [49], while the presence of DTAB with a long carbon chain enabled the nanocluster surface to be fully covered and the dissociated PPh_3_ could no longer be re-anchored onto the nanocluster vertex. Besides, the large steric hindrance of DTAB might also cause the PPh_3_ dissociation in light of the steric effect in the nanocluster crystal lattice. Indeed, the Ag_29_ nanocluster would maintain its framework, and no PPh_3_ ligand was peeled off from the cluster surface when TMAB and TBAB surfactants with short carbon chains were introduced. Of note, such a bare Ag_29_ structure has also been discovered in the presence of C_60_ with a large steric (i.e., Ag_29_(SSR)_12_(C_60_)*_n_*) [50].

Furthermore, the Ag^+^ addition to the “bare” Ag_29_ triggered the re-protection of partly exposed Ag sites (2/4; the 50% re-occupation) on the nanocluster surface, yielding the Ag_29_-Ag nanocluster (Fig. 1c and S5). Significantly, because of the presence of the combined Ag^+^, Ag_29_-Ag displayed a symmetry-breaking construction. The Ag(cluster vertex)•••Ag(combination) bond lengths were all around 2.77 Å, demonstrating its strong binding ability given that such lengths were close to the Ag(kernel)•••Ag(icosahedral surface) bond lengths in the Ag_13_ kernel of the nanocluster. The corresponding bond lengths among Ag_29_-PPh_3_, Ag_29_, and Ag_29_-Ag nanoclusters were further compared (Fig. S6). The average bond lengths of Ag(kernel)•••Ag(icosahedral surface) in Ag_29_ and Ag_29_-Ag were both lengthened relative to that of Ag_29_-PPh_3_ with 0.98% and 0.76%, respectively (Fig. S6a). Besides, the average Ag(icosahedral surface)•••Ag(icosahedral surface) bond lengths in Ag_29_-PPh_3_ were increased by 2.06% and 0.76%, respectively, to Ag_29_ and Ag_29_-Ag (Fig. S6b). In addition, the average Ag(icosahedral surface)•••Ag(motif) bond length displayed a 1.03% elongation in both Ag_29_ and Ag_29_-Ag compared with that of the Ag_29_-PPh_3_ (Fig. S6c). Accordingly, both the icosahedral Ag_13_ kernel and the Ag_25_(SSR)_12_ framework were extended with the conversion from Ag_29_-PPh_3_ to Ag_29_ and Ag_29_-Ag. As for the interactions between the Ag vertex and the icosahedral Ag_13_, the average bond lengths in Ag_29_ and Ag_29_-Ag were 3.058 and 3.151 Å, respectively (Fig. S6d, solid lines). However, no analogous interaction was perceived in Ag_29_-PPh_3_ ― distances between them (Fig. S6d, dotted lines) ranged from 3.493 to 3.643 Å (averagely, 3.523 Å). Consequently, the vertex Ag atoms became closer to the icosahedral Ag_13_ kernel upon the conversion from Ag_29_-PPh_3_ to both Ag_29_ and Ag_29_-Ag, and the newly generated Ag_4_ pyramid-like structures rendered the Ag_29_ framework more robust [51,52].

Ag_29_-PPh_3_ cluster compounds can be crystallized with cubic and trigonal systems, i.e., Ag_29_-PPh_3_-cubic and Ag_29_-PPh_3_-trigonal (Fig. S7a,b) [48,53]. Although the crystals of Ag_29_ and Ag_29_-Ag appeared to be cubes, the same as those of Ag_29_-PPh_3_ crystals, the crystalline systems of both Ag_29_ and Ag_29_-Ag were orthorhombic with space groups of *Pbcn* and *C*222_1_, respectively (Fig. S7c–e). The comparisons of cell parameters of different Ag_29_ crystal lattices were shown in Fig. S7f. Each crystal lattice of Ag_29_-Ag was composed of four cluster units, i.e., 4*{[Ag_29_(SSR)_12_-Ag_2_]_2_[Ag_29_(SSR)_12_]_1_}, and the three cluster entities in a unit were packed with a “Λ-shape” mode (Fig. S8).

**Fig. 2:**
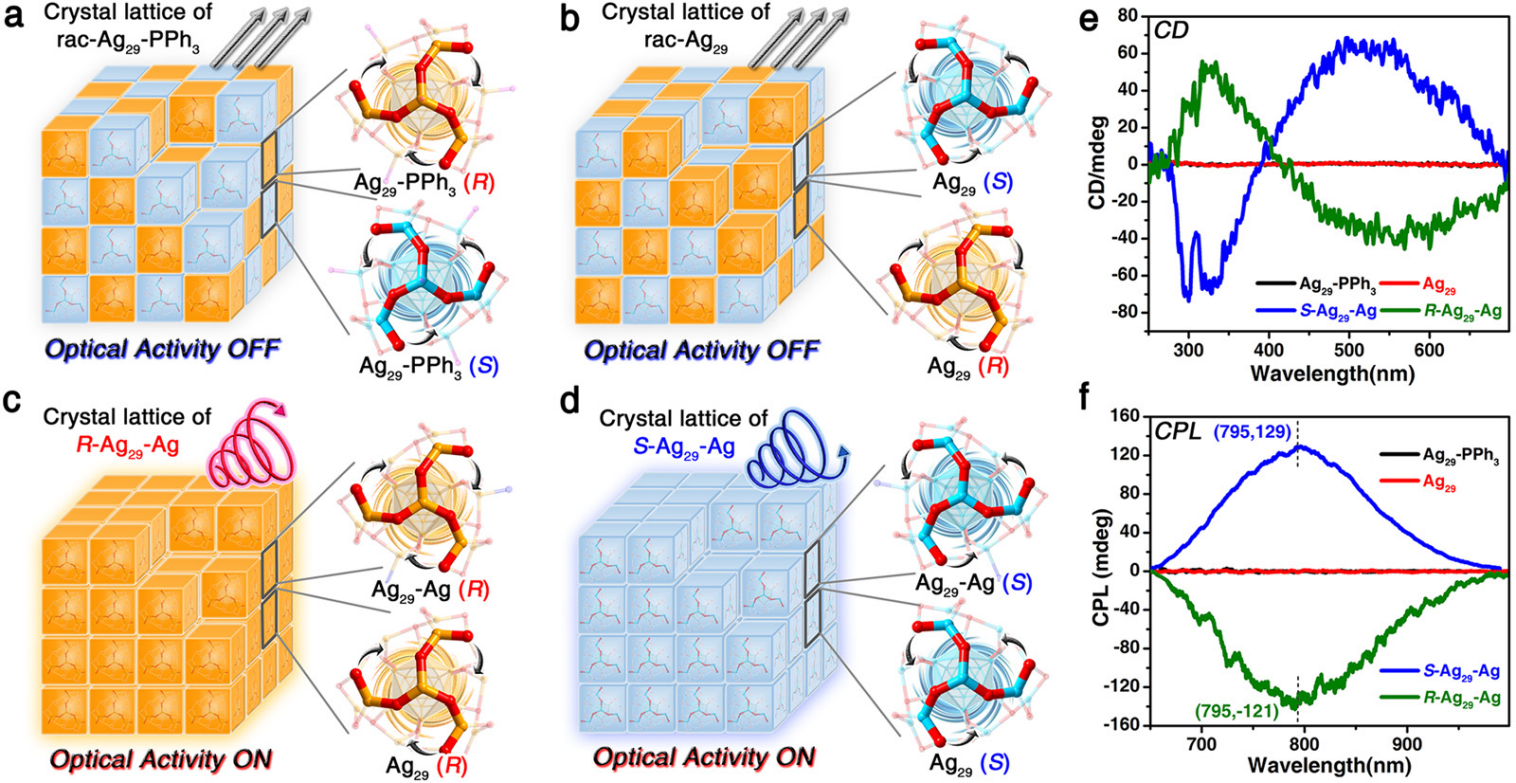
**Packing of Ag_29_ nanocluster entities in the crystal lattice and the corresponding optical activity**. (a) Crystal lattice of the racemic Ag_29_-PPh_3_ nanoclusters with no optical activity. (b) Crystal lattice of the racemic Ag_29_ nanoclusters with no optical activity. (c) Crystal lattice of chiral Ag_29_-Ag nanoclusters (*R* enantiomer) with optical activity. (d) Crystal lattice of chiral Ag_29_-Ag nanoclusters (S enantiomers) with optical activity. The orange and blue labels of Ag_29_ cluster entities represent their R and S enantiomerism, respectively. (e) CD spectra of different crystals of the Ag_29_ series. (f) CPL spectra of different crystals of the Ag_29_ series.

The innermost icosahedral Ag_13_ in the Ag_29_ nanocluster was highly symmetrical, whereas the asymmetric arrangement of the surface "triskelion"-like Ag_4_(SR)_6_ architectures rendered the chiral torsion of the overall cluster framework (Fig. 1). For Ag_29_-PPh_3_, each crystal lattice was composed of eight Ag_29_ cluster compounds, half of which were *R*-enantiomers while another half were *S*-enantiomers (Fig. 1a). In this context, the Ag_29_-PPh_3_ crystal was racemic (Fig. 2a). The same phenomenon was observed in the Ag_29_ crystal lattice (Fig. 1b and 2b). Consequently, although both Ag_29_-PPh_3_ and Ag_29_ nanocluster crystals were highly emissive, their crystals were CD and CPL silent and optically inactive (Fig. 2e,f, black and red lines).

Significantly, the Ag_29_-Ag nanocluster entities underwent chiral self-assembly with the crystallization process, which was reminiscent of the behavior of tartaric acids. Although the crystal lattice of Ag_29_-Ag contained both Ag_29_-Ag and Ag_29_ cluster molecules, all these molecules were *R*-type (or *S*-type) enantiomers in the *R*-Ag_29_-Ag crystal (or *S*-Ag_29_-Ag crystal), as depicted in Fig. 1c,d and 2c,d. In this context, the crystals of *R*-Ag_29_-Ag and *S*-Ag_29_-Ag displayed intense CD and CPL signals and were highly optically active. Luo et al. reported that the addition of ligands to superatom structures could activate or passivate a nanocluster [58]. Herein, the combination of active-Ag site exposing induced by DCTB addition and the partly re-protecting induced by Ag^+^ addition might activate the Ag_29_(SSR)_12_ cluster framework to follow an asymmetrically crystallographic packing and display highly optical activities. As shown in Fig. 2e and S9, *R*-Ag_29_-Ag and *S*-Ag_29_-Ag crystals exhibited mirror-image CD signals in the same wavelength region (i.e., at about 330 and 530 nm) with a dissymmetry factor of |*g*| = 7.0 × 10^−4^. By comparison, the enantio-separated Ag_29_-PPh_3_ nanocluster solutions displayed mirror image CD spectra at 460 nm with a dissymmetry factor of 1.5 × 10^−3^, much higher than that of the crystal of *R*-Ag_29_-Ag and *S*-Ag_29_-Ag [47]. The CPL results of *R*-Ag_29_-Ag and *S*-Ag_29_-Ag crystals showed a single signal at 795 nm (Fig. 2f). The maximum |*g*_lum_| value of *R*-Ag_29_-Ag or *S*-Ag_29_-Ag crystals was determined to be approximately 5 × 10^−2^ (Fig. S10), much higher than those of the reported metal nanoclusters [54], [55], [56], demonstrating the high optical activity of these nanocluster crystals. By comparison, the chiral Ag_29_ nanoclusters protected by DHLA (dihydrolipoic acid) exhibited mirrored CPL signals at 660 nm with |*g*_lum_| value of 2 × 10^−3^ [57]. The differences of the CD signals between enantio-separated Ag_29_-PPh_3_ solutions and chiral Ag_29_-Ag crystals as well as the CPL signals between chiral Ag_29_(DHLA)_12_ solutions and chiral Ag_29_-Ag crystals might stem from two aspects: (*i*, on the molecular level) their different molecular structures and surface chemistry and (*ii*, on the supramolecular level) their different packing states and intercluster interactions.

Upon the dissolution of the Ag_29_-Ag crystal, the CPL signal was disappeared (Fig. S11a), suggesting that the chirality tautomerism occurred in equilibrium. Inversely, the re-crystallization of the Ag_29_-Ag solution induced the chiral crystallographic self-assembly of cluster compounds that rendered the nanocluster CPL active again. Accordingly, the reversible transformation between the CPL-off solution and the CPL-on crystal of clusters has been accomplished (Fig. S11b). Because all solutions of Ag_29_-PPh_3_, Ag_29_, and Ag_29_-Ag were optically inactive, the generation of CPL was irrelevant to the PPh_3_ dissociation, but was related to the Ag^+^ combination. Besides, considering that the Ag^+^ combination on the Ag_29_(SSR)_12_ surface was not that robust (as evidenced by ESI-MS), we defined that the chiral self-assembly was mainly triggered by the Ag^+^ combination among the cluster crystallization. Of note, the Nakashima group has reported the enantiomers of the Ag_29_-PPh_3_ nanocluster by using high-performance liquid chromatography (HPLC) [47]. These Ag_29_-PPh_3_ cluster molecules maintained their structures and compositions during the HPLC process. Compared with the HPLC technology, the crystallization-induced enantioseparation in this work was assigned to a chemical approach wherein the composition and configuration of the nanocluster were altered to a certain extent.

**Fig. 3:**
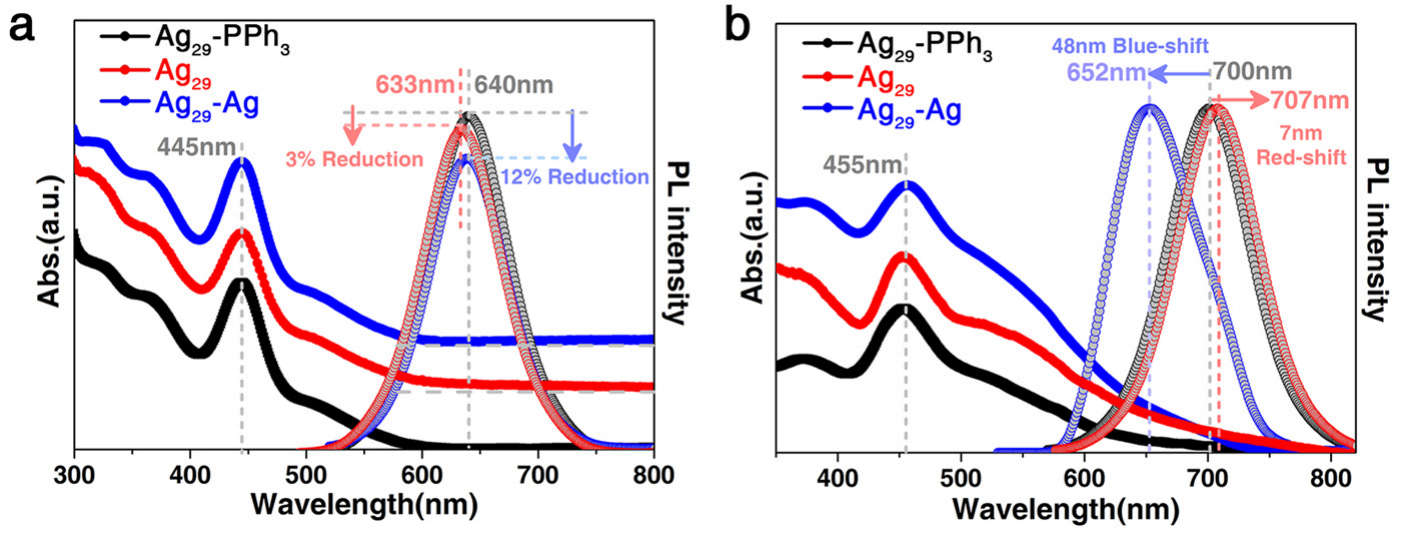
**Optical properties of different Ag_29_ nanoclusters**. (a) Comparison of optical absorptions and PL emissions (nanoclusters were dissolved in DMF) of Ag_29_-PPh_3_ (black lines), Ag_29_ (red lines), and Ag_29_-Ag (blue lines) nanoclusters. (b) Comparison of optical absorptions and PL emissions (nanoclusters were in a crystallized film) of Ag_29_-PPh_3_ (black lines), Ag_29_ (red lines), and Ag_29_-Ag (blue lines) nanoclusters.

The DMF solutions of all Ag_29_ nanoclusters exhibited almost the same optical absorptions with an intense peak at 445 nm and several shoulder bands at 320, 365, and 510 nm (Fig. 3a, solid lines); such similar absorptions demonstrated that the electronic orbits of clusters were mainly constituted by the Ag_29_(SSR)_12_ framework, but were irrelevant to the surface PPh_3_ or Ag stabilizers, which was reminiscent of the electronic properties of the Ag_29_(DHLA)_12_ nanocluster [59]. Besides, the Ag_29_-PPh_3_ and Ag_29_-Ag cluster solutions emitted at 640 nm, while the Ag_29_ displayed photoluminescence (PL) at 633 nm (Fig. 3a, dotted lines). The Ag_29_-PPh_3_ showed the strongest PL intensity among all Ag_29_ clusters, and 3% and 12% reductions were detected by comparing the emission intensities of Ag_29_ and Ag_29_-Ag, respectively, with that of Ag_29_-PPh_3_.

The optical absorptions and emissions of cluster crystalline films were further compared (Fig. 3b). The optical absorptions of all Ag_29_ films were similar, whereas the 525 nm peak of Ag_29_ was more intensive than those of other clusters (Fig. 3b, solid lines). The more pronounced absorption feature at 525 nm of the Ag_29_ crystalline film might arise due to the intercluster close packing enhancing the excitations between the Ag_13_ subunit and surface ligands [59]. Besides, a 10 nm red-shift was observed (i.e., 455 nm *versus* 445 nm) by comparing these absorptions with those of the cluster solutions. The emission wavelengths of cluster crystallized films displayed remarkable red-shifts relative to those of cluster solutions ― Ag_29_-PPh_3_ film emitted at 700 nm, Ag_29_ film emitted at 707 nm, and Ag_29_-Ag film emitted at 652 nm (Fig. 3b, dotted lines). The significant shift in emissions between different forms was expected to result from a combined effect of the electronic coupling and of lattice-origin, non-radiative decay pathways occurring through electron-phonon interactions [48,60,61]. In reference to the red-shift of emissions from the 700 nm of the Ag_29_-PPh_3_ film to the 707 nm of the Ag_29_ film, or the blue-shift to the 652 nm of the Ag_29_-Ag film, in addition to the aforementioned explanations, such a shift was also rationalized in terms of the different surface structures and crystalline packing modes among different Ag_29_ nanoclusters [51,52,62].

## 4 Conclusion

In summary, a strategy termed “active-site exposing and partly re-protecting” was presented to trigger the symmetry breaking of highly symmetrical nanoclusters and the chiral self-assembly of cluster molecules in crystals, and to render these crystals highly optically active. The introduction of counterions with large steric hindrance dissociated the PPh_3_ from the symmetrical Ag_29_(SSR)_12_(PPh_3_)_4_ nanocluster, and the vertex exposed Ag active sites of the nanoclusters was re-protected by Ag^+^, yielding an Ag_29_(SSR)_12_-Ag_2_ nanocluster with a symmetry-breaking construction. The obtained Ag_29_(SSR)_12_-Ag_2_ underwent chiral self-assembly with the crystallization process, and its crystal displayed strong optical activity, determined by both CD and CPL characterizations. Our work presents a new strategy for breaking the symmetry of highly symmetrical nanoclusters, inducing the enantioseparation of nanocluster racemates, and achieving strong optical activity of cluster-based nanomaterials.

## Declaration of competing interest

The authors declare that they have no conflicts of interest in this work.

## Appendix Supplementary materials

Image, application 1
