# Supplementary material for: Symmetry breaking of highly symmetrical nanoclusters for triggering highly optical activity
Source: Fundam Res. 2022 Mar 29;4(1):63–8. doi: 10.1016/j.fmre.2022.03.007 (PMC11197546; doi:10.1016/j.fmre.2022.03.007)
Supplement: Supplementary file 1 [file mmc1.docx]

***Supporting Information***

**Symmetry Breaking of Highly Symmetrical Nanoclusters for Triggering Highly Optical Activity**

Xiao Wei, Hao Li, Honglei Shen, Chuanjun Zhou, Shuxin Wang, Xi Kang*, Manzhou Zhu*

Department of Chemistry and Centre for Atomic Engineering of Advanced Materials, Anhui Province Key Laboratory of Chemistry for Inorganic/Organic Hybrid Functionalized Materials, Key Laboratory of Structure and Functional Regulation of Hybrid Materials, Anhui University, Ministry of Education, Anhui University, Hefei 230601, P. R. China.

* Corresponding authors: kangxi_chem@ahu.edu.cn (X.K.), zmz@ahu.edu.cn (M.Z.).

Notes: The authors declare no competing financial interest.

*This Supporting Information includes:*

Figs. S1-S11

Tables S1-S5


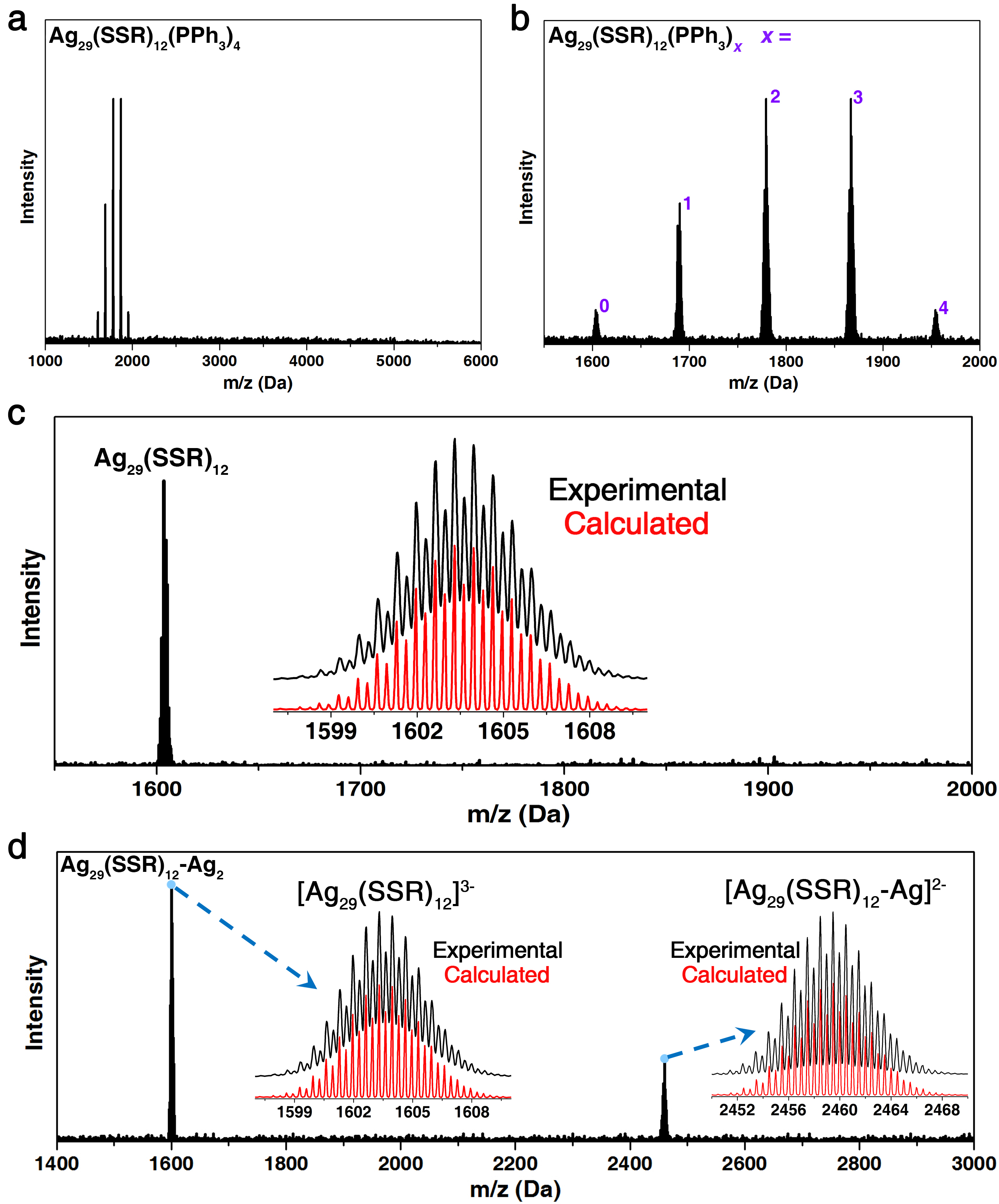


**Fig. S1. ESI-MS results of Ag_29_ nanoclusters.** (a) ESI-MS result of the Ag_29_(SSR)_12_(PPh_3_)_4_ nanocluster in a negative mode in a mass range from 1000 to 6000 Da. (b) ESI-MS result of the Ag_29_(SSR)_12_(PPh_3_)_4_ nanocluster in a negative mode in a mass range from 1500 to 2000 Da. Several mass signals were observed, corresponding to [Ag_29_(SSR)_12_(PPh_3_)*_x_*]^3-^ (*x* = 0-4). (c) ESI-MS result of the Ag_29_(SSR)_12_ nanocluster in a negative mode in a mass range from 1500 to 2000 Da. (d) ESI-MS result of the Ag_29_(SSR)_12_-Ag_2_ nanocluster in a negative mode in a mass range from 1400 to 3000 Da. Two mass signals were observed, corresponding to [Ag_29_(SSR)_12_]^3-^ and [Ag_29_(SSR)_12_-Ag]^2-^. However, the mass peak of [Ag_29_(SSR)_12_-Ag_2_]^1-^ was absent, which might result from the weak interactions between the Ag_29_(SSR)_12_ framework and Ag^+^ ions. Of note, the intensity of the [Ag_29_(SSR)_12_]^3-^ mass signal was much higher than that of [Ag_29_(SSR)_12_-Ag]^2-^. Some of these detected bare Ag_29_(SSR)_12_ molecules should be generated from the dissociation of Ag_29_(SSR)_12_-Ag_2_, which partly verified the weak interaction between Ag_29_(SSR)_12_ and Ag^+^.


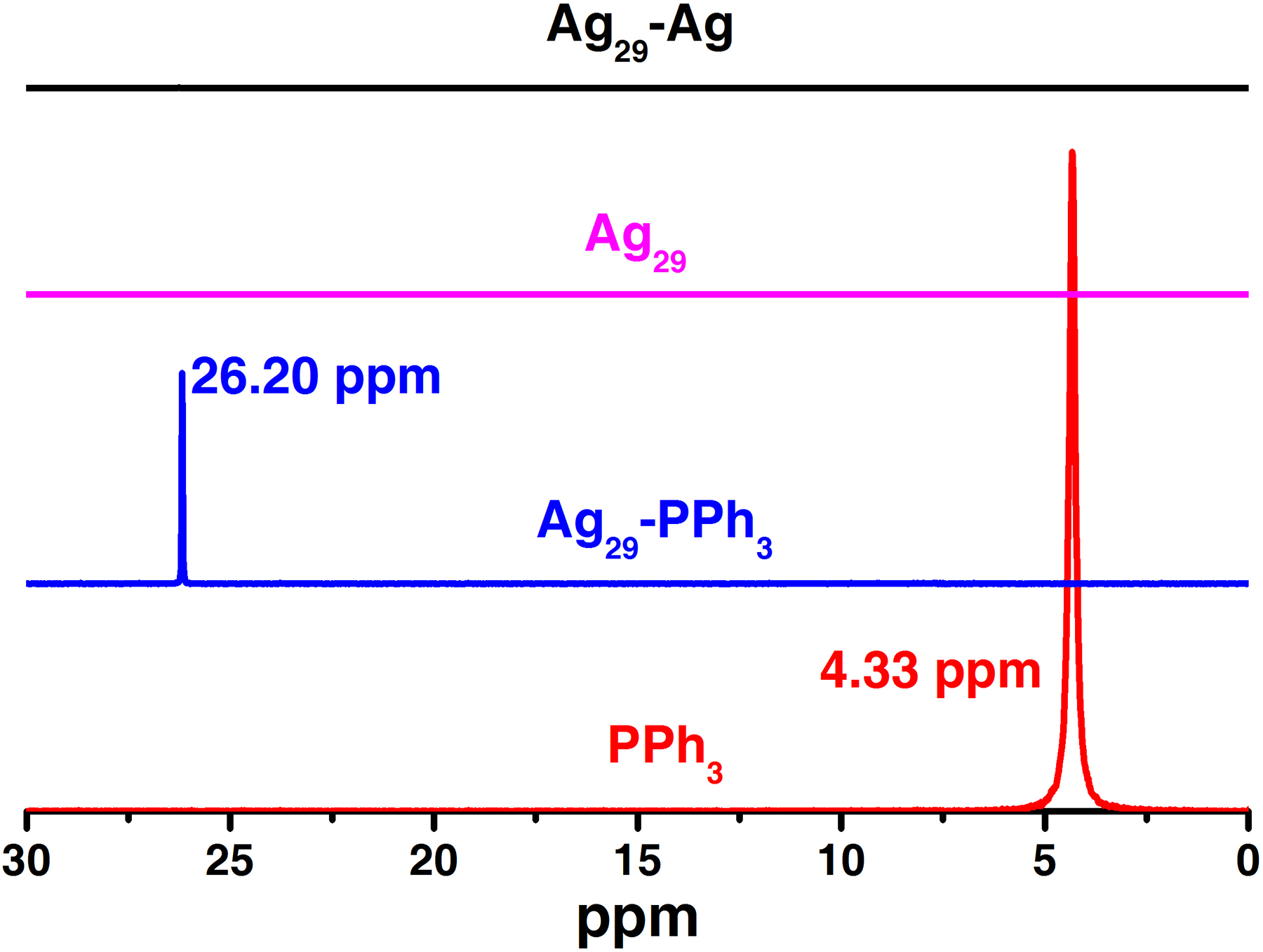


**Fig. S2.** **^31^P NMR results of PPh_3_ (red), Ag_29_-PPh_3_ (blue), Ag_29_ (magenta), and Ag_29_-Ag (black) nanoclusters.** Nanoclusters were dissolved in deuterated DMF and then the ^31^P NMR measurement was performed.


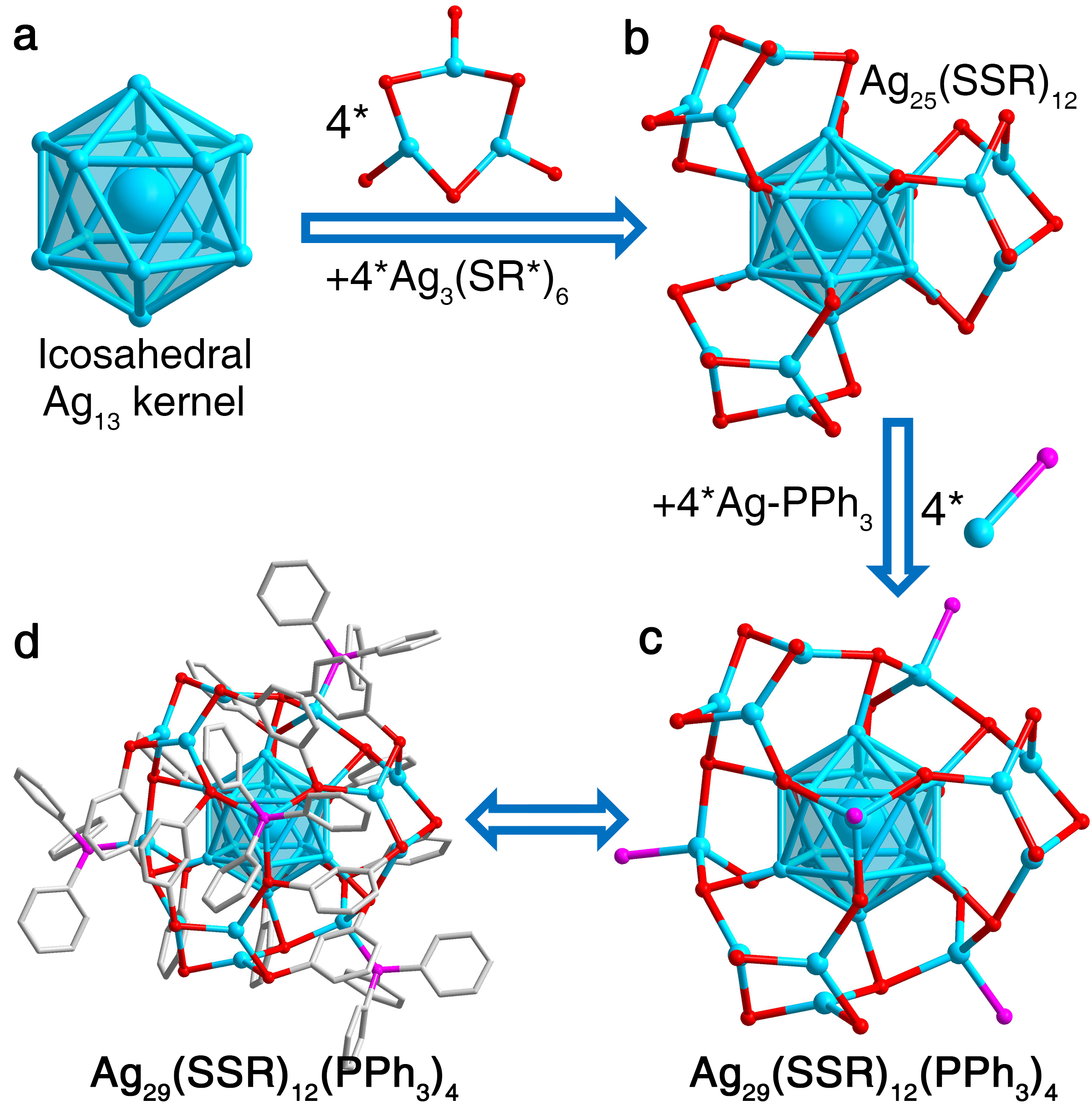


**Fig. S3. Structural anatomy of the Ag_29_(SSR)_12_(PPh_3_)_4_ nanocluster.** (a) The icosahedral Ag_13_ kernel. (b) The Ag_25_(SSR)_12_ structure containing an Ag_13_ kernel and six Ag_3_(SR^*^)_6_ motif structures. The SR^*^ represents half of the SSR ligand. (c) The Ag_29_(SSR)_12_(PPh_3_)_4_ framework containing the Ag_25_(SSR)_12_ structure and four Ag-PPh_3_ vertex units. (d) The overall structure of the Ag_29_(SSR)_12_(PPh_3_)_4_ nanocluster. Color legends: light blue sphere, Ag; red sphere, S; magenta sphere, P; grey sphere, C. For clarity, all H atoms are omitted.


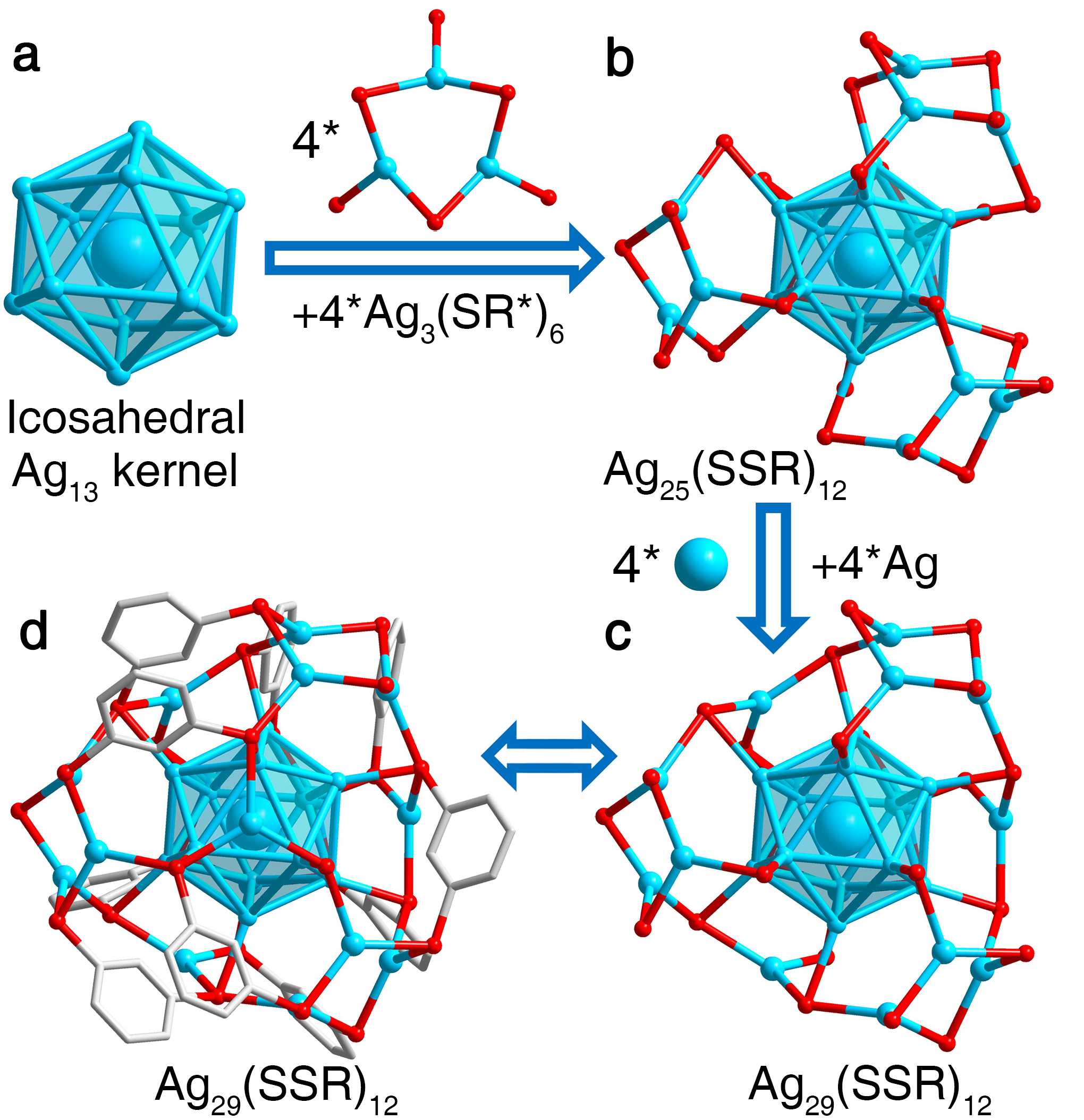


**Fig. S4.** **Structural anatomy of the Ag_29_(SSR)_12_ nanocluster.** (a) The icosahedral Ag_13_ kernel. (b) The Ag_25_(SSR)_12_ structure containing an Ag_13_ kernel and six Ag_3_(SR^*^)_6_ motif structures. The SR^*^ represents half of the SSR ligand. (c) The Ag_29_(SSR)_12_(PPh_3_)_4_ framework containing the Ag_25_(SSR)_12_ structure and four Ag vertex atoms. (d) The overall structure of the Ag_29_(SSR)_12_ nanocluster. Color legends: light blue sphere, Ag; red sphere, S; magenta sphere, P; grey sphere, C. For clarity, all H atoms are omitted.


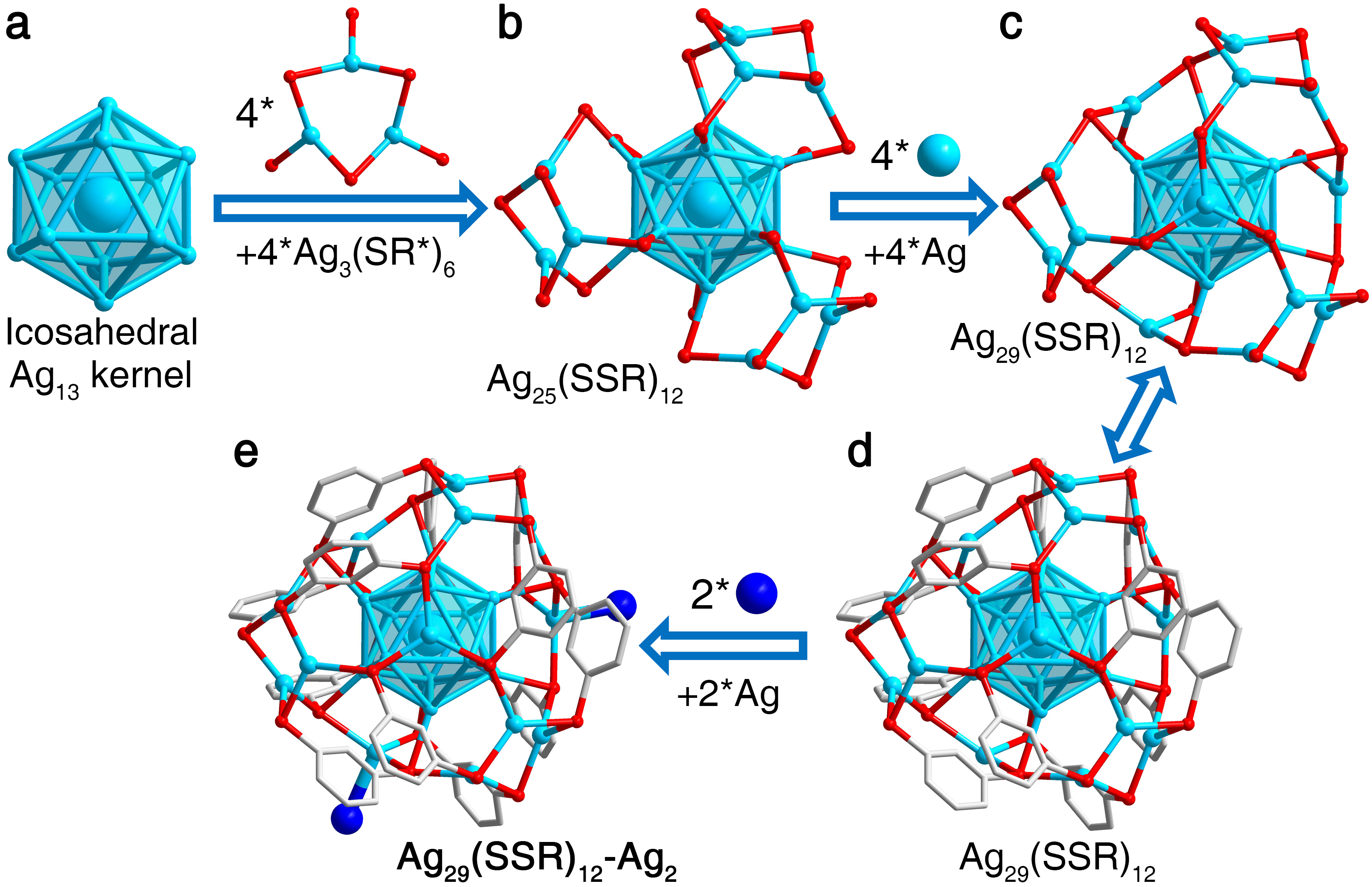


**Fig. S5.** **Structural anatomy of the Ag_29_(SSR)_12_-Ag_2_ nanocluster.** (a) The icosahedral Ag_13_ kernel. (b) The Ag_25_(SSR)_12_ structure containing an Ag_13_ kernel and six Ag_3_(SR^*^)_6_ motif structures. The SR^*^ represents half of the SSR ligand. (c,d) The Ag_29_(SSR)_12_(PPh_3_)_4_ framework containing the Ag_25_(SSR)_12_ structure and four Ag vertex atoms. (e) The overall structure of the Ag_29_(SSR)_12_-Ag_2_ nanocluster containing the Ag_29_(SSR)_12_(PPh_3_)_4_ framework and two anchoring Ag atoms. Color legends: blue/light blue sphere, Ag; red sphere, S; magenta sphere, P; grey sphere, C. For clarity, all H atoms are omitted.


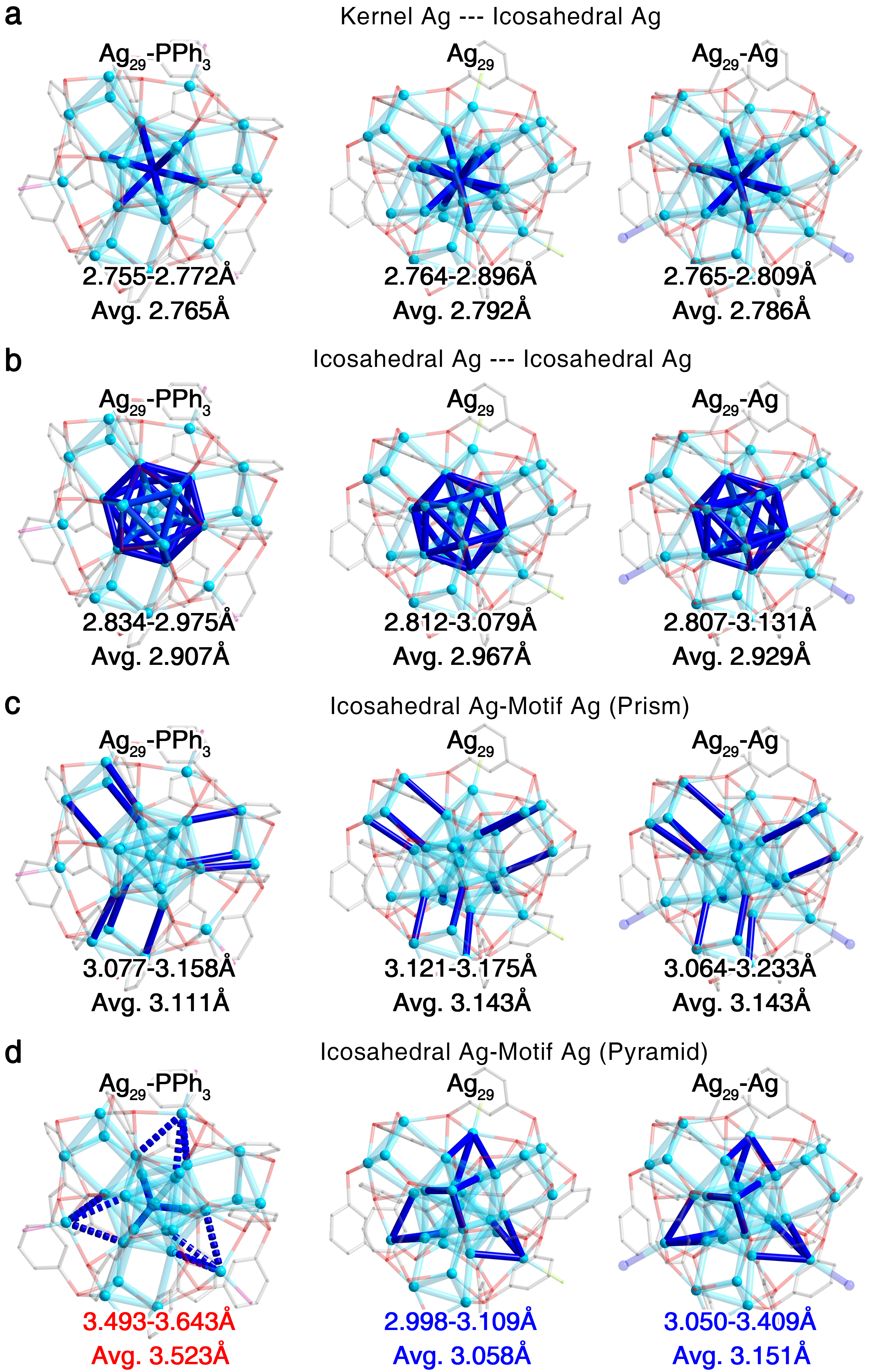


**Fig. S6.** **Comparison of corresponding bond lengths among Ag_29_(SSR)_12_(PPh_3_)_4_, Ag_29_(SSR)_12_, and Ag_29_(SSR)_12_-Ag_2_ nanoclusters.** (a) Comparison of the length of Ag(kernel)-Ag(icosahedral surface) bonds. (b) Comparison of the length of Ag(icosahedral surface)-Ag(icosahedral surface) bonds. (c) Comparison of the length of prism-like Ag(icosahedral surface)-Ag(motif) bonds. (d) Comparison of the length of pyramid-like Ag(icosahedral surface)-Ag(motif) bonds.


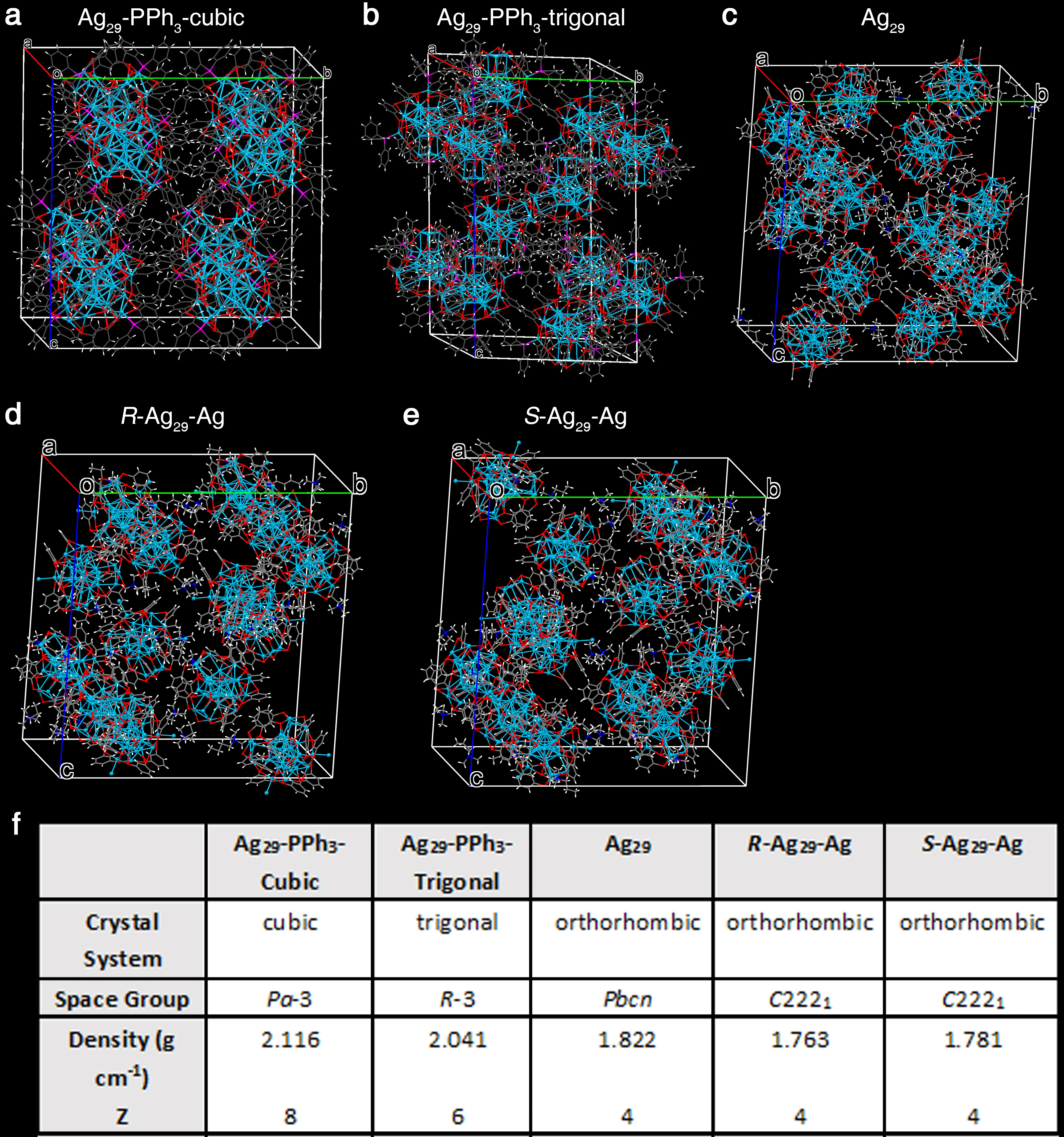


**Fig. S7.** **Comparison of crystal lattices of Ag_29_ nanoclusters.** (a) Crystal lattice of Ag_29_(SSR)_12_(PPh_3_)_4_ with a cubic crystal system. (b) Crystal lattice of Ag_29_(SSR)_12_(PPh_3_)_4_ with a trigonal crystal system. (c) Crystal lattice of Ag_29_(SSR)_12_. (d) Crystal lattice of *R*-Ag_29_(SSR)_12_-Ag_2_. (e) Crystal lattice of *S*-Ag_29_(SSR)_12_-Ag_2_. (f) Comparison of cell parameters of different crystal lattices of the Ag_29_ series.


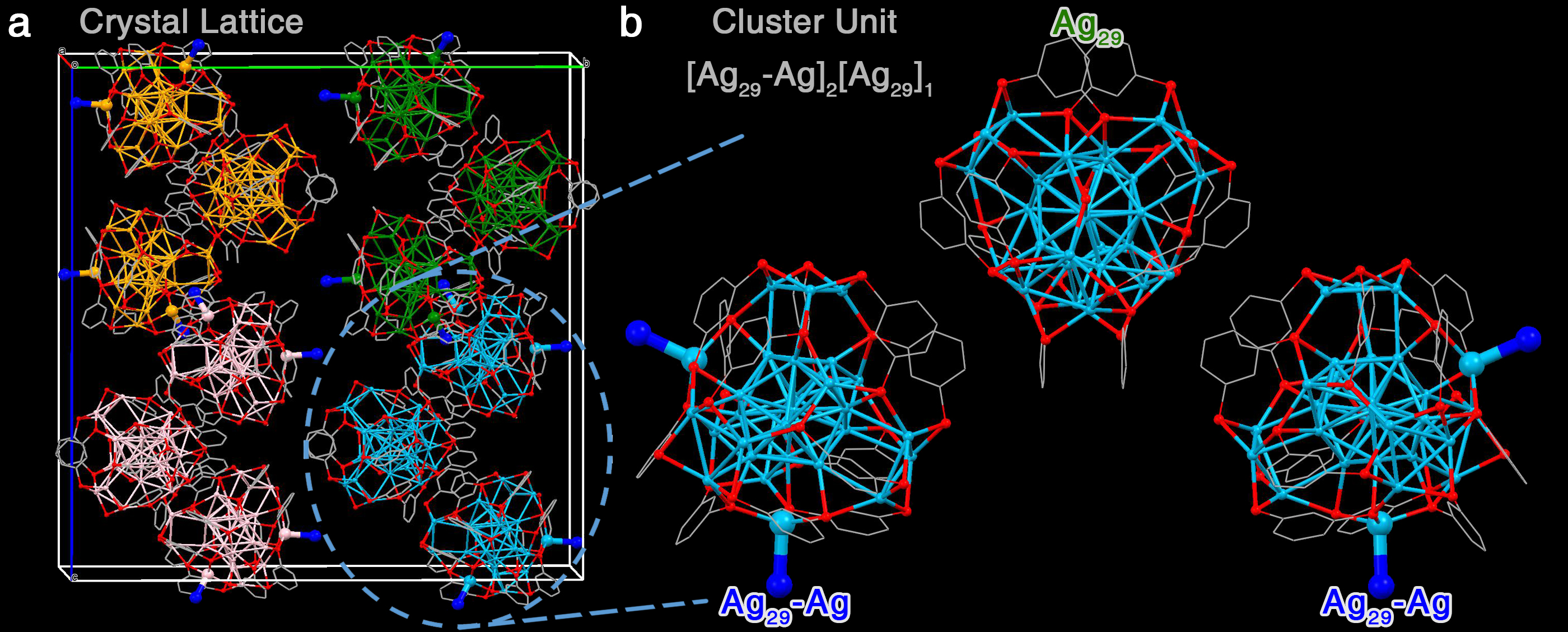


**Fig. S8.** **Crystal lattice of Ag_29_(SSR)_12_-Ag_2_ and Ag_29_(SSR)_12_ nanoclusters.** (a) Distributions of Ag_29_(SSR)_12_-Ag_2_ and Ag_29_(SSR)_12_ nanoclusters in the crystal lattice. (b) The molecular ratio between Ag_29_(SSR)_12_-Ag_2_ and Ag_29_(SSR)_12_ nanoclusters in the crystal lattice was 2:1.


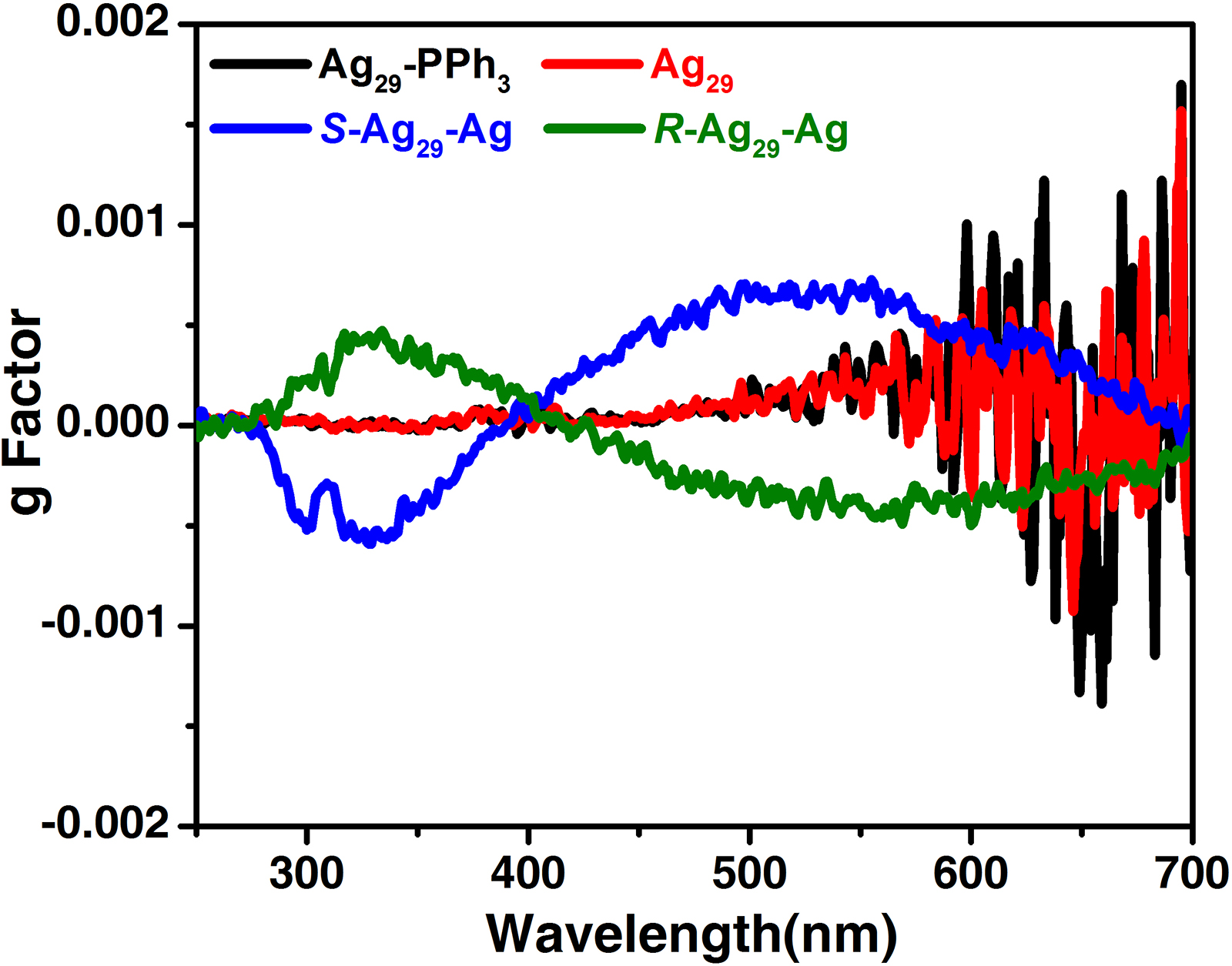


**Fig. S9.** ***g* factors of the CD results (corresponding to Fig. 2e) of different crystals of the Ag_29_ series.**


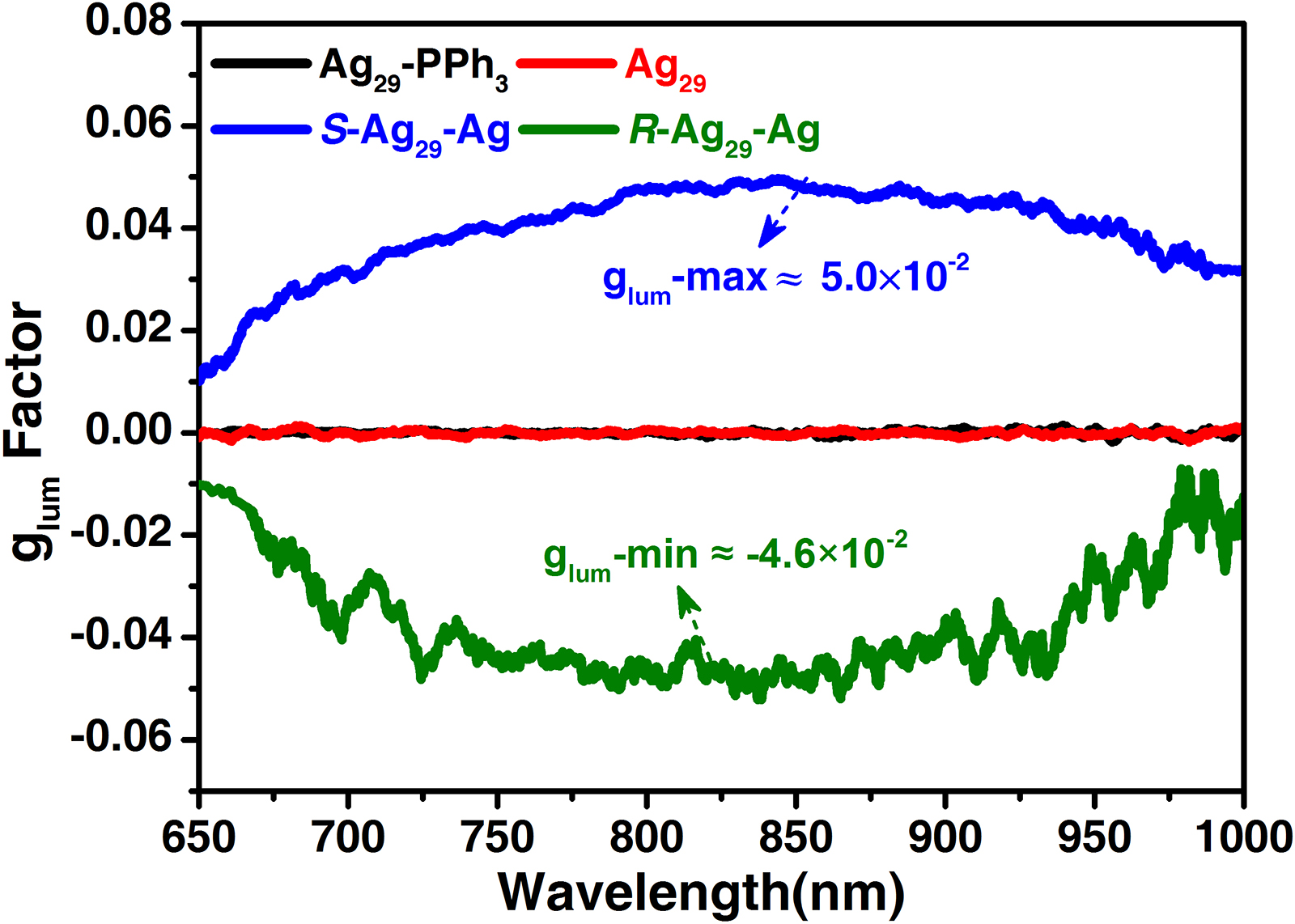


**Fig. S10.** ***g_lum_* factors of the CPL results (corresponding to Fig. 2f) of different crystals of the Ag_29_ series.**


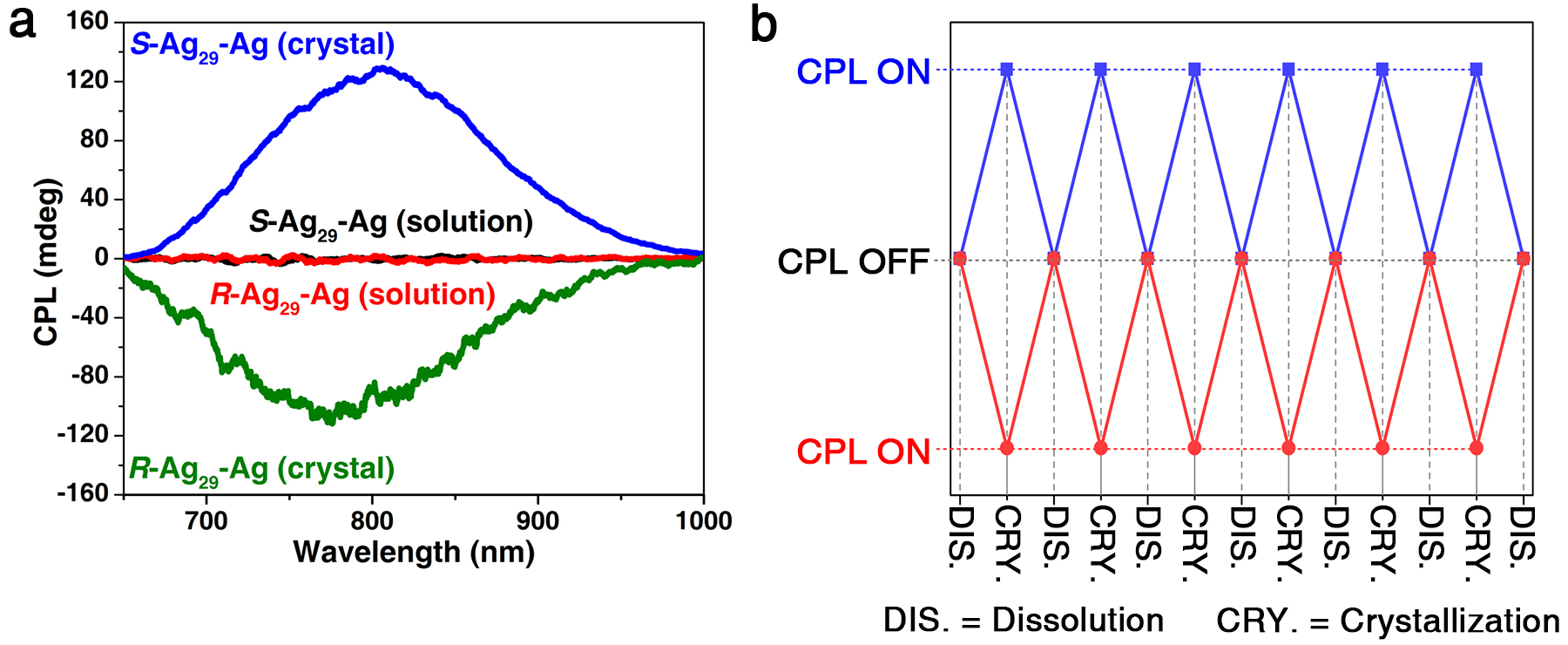


**Fig. S11.** **CPL results of Ag_29_ nanoclusters in solutions and crystals.** (a) CPL results of the chiral Ag_29_(SSR)_12_-Ag_2_ and Ag_29_(SSR)_12_ nanocluster crystals (*S* or *R* enantiomers, labeled in blue and green, respectively). CPL results of the chiral Ag_29_(SSR)_12_-Ag_2_ and Ag_29_(SSR)_12_ nanocluster solutions (*S* or *R* enantiomers, dissolved in DMF, labeled in black and red, respectively). (b) Reversible conversion between the Ag_29_-Ag in the crystalline state with CPL and the Ag_29_-Ag in the solution state without CPL. The crystallization operation of the Ag_29_-Ag solution induces the chiral self-assembly of cluster compounds in the crystal lattice that the cluster crystals displayed the CPL. Of note, after each crystallization, half of the crystals are *R*-Ag_29_(SSR)_12_-Ag_2_ while another half are *S*-Ag_29_(SSR)_12_-Ag_2_. The dissolution operation of the Ag_29_-Ag crystal resulted in the solution of racemic clusters with no CPL signal.

**Table S1.** **Crystal data and structure refinement for the racemic Ag_29_(SSR)_12_.** The CCDC number is 2071574.

| Crystal system | orthorhombic |
| --- | --- |
| Space group | P b c n |
| a/Å | 24.9062(7) |
| b/Å | 45.3226(11) |
| c/Å | 48.2123(13) |
| α/° | 90 |
| β/° | 90 |
| γ/° | 90 |
| Volume/Å^3^ | 54423(2) |
| Z | 4 |
| ρcalcg/cm^3^ | 1.822 |
| μ/mm^‑1^ | 27.225 |
| F(000) | 27860 |
| Radiation | CuKα (λ = 1.54186) |
| Index ranges | -29 ≤ h ≤ 29, -54 ≤ k ≤ 54, -15 ≤ l ≤ 56 |
| θ range (°) | 3.371 – 69.711 |
| Measured reflections and unique reflections | 267117 / 49846 (*R*_int_ = 0.0791) |
| Goodness-of-fiton F^2^ | 1.074 |
| Largest diff. peak/hole / e Å^-3^ | 2.2/-1.5 |
| Final R indexes [I>=2σ (I)] | R1 = 0.1186, wR2 = 0.2509 |
| Final R indexes [all data] | R1 = 0.1364, wR2 = 0.2411 |

**Table S2.** **Crystal data and structure refinement for the chiral Ag_29_(SSR)_12_-Ag_2_ and Ag_29_(SSR)_12_ nanoclusters (*R* enantiomers).** The CCDC number is 2071643.

| Crystal system | orthorhombic |
| --- | --- |
| Space group | C 2 2 2_1_ |
| a/Å | 24.947(2) |
| b/Å | 47.041(2) |
| c/Å | 49.157(4) |
| α/° | 90 |
| β/° | 90 |
| γ/° | 90 |
| Volume/Å^3^ | 57688.0(3) |
| Z | 4 |
| ρcalcg/cm^3^ | 1.763 |
| μ/mm^‑1^ | 26.223 |
| F(000) | 28668 |
| Radiation | CuKα (λ = 1.54186) |
| Index ranges | -18 ≤ h ≤ 30, -53 ≤ k ≤ 56, -59 ≤ l ≤ 47 |
| θ range (°) | 4.68 – 70.56 |
| Measured reflections and unique reflections | 262688 / 52843 (*R*_int_ =0.0683) |
| Goodness-of-fiton F^2^ | 0.995 |
| Largest diff. peak/hole / e Å^-3^ | 2.5/-1.2 |
| Final R indexes [I>=2σ (I)] | R1 = 0.0581, wR2 = 0.1534 |
| Final R indexes [all data] | R1 = 0.0668, wR2 = 0.1479 |

There are two level A alerts in this crystal:

PLAT308_ALERT_2_A Single Bonded Metal Atom in Structure (Unusual) Ag46 Check;

PLAT308_ALERT_2_A Single Bonded Metal Atom in Structure (Unusual) Ag47 Check.

The two level A alerts result from the two anchored Ag atoms on the nanocluster surface, which are singly bonded with the surface Ag of the Ag_29_ kernel. Actually, there are still weak interactions between the anchored Ag atoms and DTAB counterions; in other words, there are several DTAB counterions in the crystal lattice surrounding the anchored Ag atoms, keeping these Ag atoms stable. However, the SC-XRD does not detect weak interactions and reports two level A alerts. On this issue, the ESI-MS measurement has been performed to confirm the presence of the anchored Ag atoms (see Fig. S1).

**Table S3.** **Crystal data and structure refinement for the chiral Ag_29_(SSR)_12_-Ag_2_ and Ag_29_(SSR)_12_ nanoclusters (*S* enantiomers).** The CCDC number is 2071575.

| Crystal system | orthorhombic |
| --- | --- |
| Space group | C 2 2 2_1_ |
| a/Å | 24.9563(4) |
| b/Å | 46.9721(6) |
| c/Å | 49.1198(8) |
| α/° | 90 |
| β/° | 90 |
| γ/° | 90 |
| Volume/Å^3^ | 57580.7(15) |
| Z | 4 |
| ρcalcg/cm^3^ | 1.781 |
| μ/mm^‑1^ | 26.278 |
| F(000) | 28924 |
| Radiation | CuKα (λ = 1.54186) |
| Index ranges | -21 ≤ h ≤ 30, -56 ≤ k ≤ 55, -43 ≤ l ≤ 59 |
| θ range (°) | 3.56 – 66.34 |
| Measured reflections and unique reflections | 292728 / 53686 (*R*_int_ =0.0523) |
| Goodness-of-fiton F^2^ | 0.980 |
| Largest diff. peak/hole / e Å^-3^ | 1.5/-2.8 |
| Final R indexes [I>=2σ (I)] | R1 = 0.0940, wR2 = 0.2653 |
| Final R indexes [all data] | R1 = 0.1097, wR2 = 0.2285 |

There are two level A alerts in this crystal:

PLAT308_ALERT_2_A Single Bonded Metal Atom in Structure (Unusual) Ag1 Check;

PLAT308_ALERT_2_A Single Bonded Metal Atom in Structure (Unusual) Ag2 Check.

The two level A alerts result from the two anchored Ag atoms on the nanocluster surface, which are singly bonded with the surface Ag of the Ag_29_ kernel. Actually, there are still weak interactions between the anchored Ag atoms and DTAB counterions; in other words, there are several DTAB counterions in the crystal lattice surrounding the anchored Ag atoms, keeping these Ag atoms stable. However, the SC-XRD does not detect weak interactions and reports two level A alerts. On this issue, the ESI-MS measurement has been performed to confirm the presence of the anchored Ag atoms (see Fig. S1).

**Table S4.** **Crystal data and structure refinement for** **Ag_29_(SSR)_12_(PPh_3_)_4_ in the presence of tetramethylammonium bromide.** The CCDC number is 2150072.

| Crystal system | trigonal |
| --- | --- |
| Space group | R -3 |
| a/Å | 27.0934(17) |
| b/Å | 27.0934(17) |
| c/Å | 47.336(3) |
| α/° | 90 |
| β/° | 90 |
| γ/° | 120 |
| Volume/Å^3^ | 30092(4) |
| Z | 6 |
| ρcalcg/cm^3^ | 1.969 |
| μ/mm^‑1^ | 25.166 |
| F(000) | 16624 |
| Radiation | CuKα (λ = 1.54186) |
| Index ranges | -15 ≤ h ≤ 32, -32 ≤ k ≤ 27, -46 ≤ l ≤ 57 |
| θ range (°) | 4.206 –69.597 |
| Measured reflections and unique reflections | 94771 / 12470 (*R*_int_ = 0.0680) |
| Goodness-of-fiton F^2^ | 0.930 |
| Largest diff. peak/hole / e Å^-3^ | 2.169 / -1.842 |
| Final R indexes [I>=2σ (I)] | *R_1_* = 0.0372, *wR_2_* = 0.0919 |
| Final R indexes [all data] | *R_1_* = 0.0511, *wR_2_* = 0.0881 |

**Table S5.** **Crystal data and structure refinement for Ag_29_(SSR)_12_(PPh_3_)_4_ in the presence of tetrabutylammonium bromide.** The CCDC number is 2150121.

| Crystal system | trigonal |
| --- | --- |
| Space group | R -3 |
| a/Å | 27.1531(9) |
| b/Å | 27.1531(9) |
| c/Å | 49.1230(19) |
| α/° | 90 |
| β/° | 90 |
| γ/° | 120 |
| Volume/Å^3^ | 31366(2) |
| Z | 6 |
| ρcalcg/cm^3^ | 1.989 |
| μ/mm^‑1^ | 2.957 |
| F(000) | 17880 |
| Radiation | MoK\a (λ = 0.71073) |
| Index ranges | -33 ≤ h ≤ 30, -21 ≤ k ≤ 34, -50 ≤ l ≤ 63 |
| θ range (°) | 2.25 – 27.01 |
| Measured reflections and unique reflections | 30063 / 16054 (*R*_int_ = 0.0369) |
| Goodness-of-fiton F^2^ | 1.048 |
| Largest diff. peak/hole / e Å^-3^ | 4.486/ -1.589 |
| Final R indexes [I>=2σ (I)] | *R_1_* = 0.0579, *wR_2_* = 0.1907 |
| Final R indexes [all data] | *R_1_* = 0.0983, *wR_2_* = 0.1628 |
